# Food for mood: Relevance of nutritional Omega-3 fatty acids for depression and anxiety

**DOI:** 10.1192/j.eurpsy.2021.98

**Published:** 2021-08-13

**Authors:** S. Laye

**Affiliations:** Inrae, Univ Bordeaux, NutriNeuro, Bordeaux, France

**Keywords:** omega-3, synaptic plasticity, neuroinflammation, depression, anxiety, microglia

## Abstract

Polyunsaturated fatty acids (PUFAs) are essential fatty acids which are provided to the body through the diet. The brain is one of the richest organs in the body and has a high need in PUFAs. There are 2 main families of PUFAs, n-3 (or omega 3) and n-6 (or omega6). While it is quite easy to find n-6 PUFAs in westernized diets, the need in n-3 PUFAs is poorly reached, leading to decreased level of docosahexaenoic acid (DHA) in the brain. In humans, poor levels of blood n-3 PUFAs and brain DHA are associated to a higher prevalence of cognitive disorders and depression. However, the mechanisms underlying the effect of DHA on brain functions are poorly understood. Using mice models of n-3 PUFAs dietary deficiency or supplementation, we revealed that in the brain, DHA regulate neuroinflammatory pathways, in particular through its effect on microglia, the main innate immune system cell in the brain. In addition, n-3 PUFAs are key actors of ndocannabinoid- dependent synaptic plasticity. While neuroinflammation and eCB-dependent synaptic plasticity are crucial to cognition and emotional behaviour alterations, our results bring to the clinical scene the importance of controlling dietary n-3 PUFAs to protect the brain from the adverse effect of stres or inflammation. Altogether, our work brings a better comprehension of how dietary n-3 PUFAs participate to brain physiology and protect from the development of mood and cognitive disorders. It opens new avenues for the use of these lipids in the protection and treatment of brain diseases.

**Disclosure:**

No significant relationships.

